# An Advanced ICTVSS Model for Real-Time Vehicle Traffic Applications

**DOI:** 10.3390/s19194134

**Published:** 2019-09-24

**Authors:** You-Shyang Chen, Chien-Ku Lin, Yao-Wen Kan

**Affiliations:** 1 Department of Information Management, Hwa Hsia University of Technology, New Taipei City 235, Taiwan; aa7885@ntpc.gov.tw; 2 Department of Information Management, National Yunlin University of Science and Technology, Yunlin 640, Taiwan; g9923808@yuntech.edu.tw

**Keywords:** real-time, pattern recognition, license plate, differential algorithm, contour algorithm

## Abstract

From the accident news, it is found that the occurrences of traffic accidents every year and the numbers of deaths and injuries have raised continually and have become a specific issue concerned in society in Taiwan. More seriously, the number of traffic accidents is positively increased with the increasing motorized vehicles. Thus, to reduce the incidence of traffic accidents through by some advanced real-time technologies is an important and interesting work. However, some serious problems against traffic safety are facing, such as the low-quality video saved by a camera, low efficiency facilities supported, inefficient management of surveillance resources, and low definition resolution for cameras, which is resulted in a dilemma problem caused from providing evidence-based images to a local authority either for criteria for judgment or basis for improvement. As a big effort to deal with the above defects for constructing a smart city, this paper makes a main purpose to develop an advanced system of intelligent cloud-based transportation vehicle surveillance (called ICTVSS) for license plate identification. This existing identification algorithm was studied and developed from a combination of improved differential algorithm and improved active contour algorithm. Given such a combination, a novel algorithm of dynamic license identification for smart monitoring was fully realized for constructing a well-defined smart city. The experimental results showed good performance and experienced that the proposed algorithm performed well in locating multi-license plate and differential methods, removing image noise of license plate, and processing constant-inconstant light source from complex environment cases, and guaranteed effective license plate identification from the benefit of high resolutions of digital cameras.

## 1. Introduction

One of the main factors of economic prosperity is convenient transportation. Due to the significant increase in vehicles, it may lead to traffic accidents more and more, such as drinking and driving, speeding, traffic violations, etc. Based on the report of National Police Agency, Ministry of the Interior, Republic of China, Taiwan (2019) [1], second highest of 2894 were killed in traffic accidents involving motorized vehicles in 2005, and the highest number was a staggering 3140 in 2006. Table 1 shows the statistical data of causes and casualties of road traffic accidents from 2003 to 2018 extracted from [1], and the shading presents its importance in the corresponding column. The financial expenses of manpower resources and medical supports were rising extremely based on Table 1. When deeply exploring the key cause from Table 1, it is found that most of automobile (motorcycle and slow-moving vehicle) driver faults is main case. Moreover, other problems are addressed for this fatal failure event, including the inefficient equipment provided and poor-quality videos recorded in the surveillance cameras hosted by local authorities and administrations, low camera definition, and inefficient management, which result in serious difficulties for giving evidence-based records for local governments either for corroborating data of justification or for supporting a basis of future improvement. As a comparison, the continuing number of traffic-related accidents is significant enhancement for negative effects. In particular, it has a warning function that uses control strategies with cameras for useful to influence the driving style to drivers from the study of [2]; therefore, it also has an emergent consciousness to design a hybrid system with cameras in using effective control ways or some useful tools or/and new techniques to lower the cases of traffic-related deaths and injuries. 

Over recent decades, digital cameras had prompted main booms from the late 1990s to the early 2000s and facilitated this progress based on accelerating technological developments; combining functionality of camera phones and digital cameras, the accessibility of smart camera significantly triggered with the demand [3]. From the study in Shi and Lichman [4], it is indicated that smart cameras are a valuable resource to perform a major activity for recording and monitoring a characterized, duplicated, high-speed, and high-quality performance of higher resolution work to learn a system for intelligent video surveillance (IVS) [5,6,7] for some advancements of the most popular camera applications in practice. Such an IVS system services a wide domain to track and detect every material’s movement (including persons), and to analyze this movement for its behavior to increase person’s safety and security in real-life applications; thus, the smart cameras have numerously extensive uses, such as traffic management of transportation vehicles, surveillance, and security enforcement [8]. For example, Stahlschmidt [9] detected individuals from a dense crowd place by learning the IVS system of smart camera applications. In recent, one of the most common applications from the view of traffic surveillance is to recognize license plate of vehicles [10,11,12]. Moreover, because of a continued improve in demand for conforming to primary function of the IVS and charge coupled device (CCD) for smart cameras [13], the state-of-the-art technology applications associated with monitoring transport vehicles have made great strides over time. In particular, modeling and integrating difference algorithm and contour algorithm of IVS systems for vehicles is a relative and new functionality. 

Furthermore, given the review of limited literatures, most studies aimed on researching and improving the gap of related techniques of image processing for the digital cameras, but it is rarely highlighted to construct a suitable smart city of using emerged image processing technologies in order to reduce risks of traffic accident and criminal cases for a developing city. Thus, good uses of emerged image processing technologies to bridge this gap on the smart city has an interesting and important topic from practical and academic perspectives; moreover, such a main purpose triggers this paper. This paper has a key purpose to design accordingly a framework with practical application of smart cities for modeling an intelligent cloud-based transportation vehicle surveillance system (ICTVSS) to complete such an issue. Three main objectives in this paper are defined as follows: (1) To develop an easily and good using vehicle surveillance system model for the prototype of the modern smart city; (2) to provide the cloud-based monitor system used to well-formed extract useful information; and (3) to provide a database of large license plate recognitions under the context of big data analysis for future traffic safety and urban management purposes. 

The rest of this paper is organized into the following presentation for the clarity of information presented. Section 2 describes an extensive literature review within the mechanism of the subject of building smart city. Thus, the architecture of the smart vehicle surveillance system is proposed in Section 3, and the proposed system is introduced with experiencing a real case. Conclusions with an insight and discussion to future improvements of potentials are reported in Section 4, Section 5 and Section 6.

## 2. Literature Review

This section reviews some references from the background knowledge of the study subject for addressing a related smart city issue, including introducing the smart city and the IVS and CCD with applications. 

### 2.1. The Smart City

A framework of smart cities for movement of road safety for citizens from an intelligent-design perspective is to employ a variety of emerging technologies, such as digital cameras and electronic sensors (e.g., IVS and CCD, respectively), to gather and measure data in order to monitor and supply information of road traffics and transportation systems efficiently. Smart technologies can monitor what is happening for handling traffic incidents and accidents in a city with reaction mechanism for city officials to promote the service efficiency of transportation operations to citizens. In addition, applications of smart technologies for traffics are to use intelligent techniques to manage urban flows and respond for real-time communications. Thus, the smart city benefits from more preparation to response to emerging problems and challenges from emerged accidents for its citizens.

Through the concept of smart city, two basic social behaviors, including territoriality and natural surveillance, are strengthened to aroused citizens to be aware of possible criminal activities and denounce them from time to time; thus, enabling the safety control of physical environment can be concerned, participated in, and monitored by residents. In terms of the function of smart city, the most urgent problem to be solved is how to cut off the lifeblood of criminals: Criminal tools, which can deter criminals from committing crimes. In the course of or before or after the crime, the car and the locomotive on the road are under the control of the electronic police force at any time; neither the committed crime nor the current crime will be able to escape. Therefore, besides setting up multi-function digital license plate recognition system at all important intersections, the vehicle-mounted and police mobile digital license plate recognition system can be used [14]. By taking a proactive approach to the deep lanes and blind angles of public order, the police can comprehensively attack and seize the specific vehicles (the main object is stolen vehicles), thereby enhancing the overall road safety environment for citizens. 

**Figure 1:**
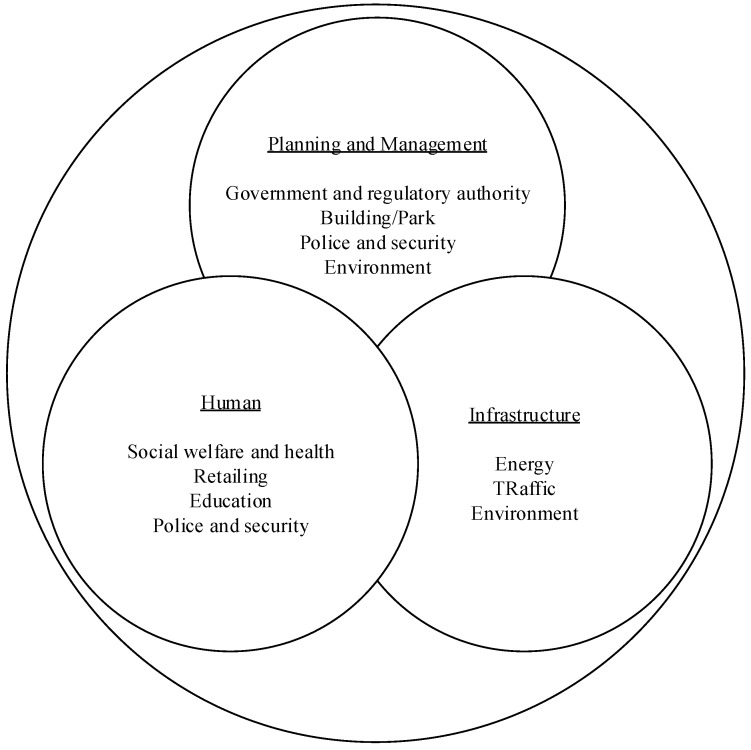
A schematic diagram of smart city.

Traffic video image analysis and planning, by integrating the abundant information service projects provided by the digital cloud platform, can help promote the strengthening of public order and rectify the service dispatch of relevant units of transportation. It is in line with the ideal of using modern scientific instruments to search traffic irregularities and achieve the effect of maintaining public order and eliminating crimes from invisible; therefore, it is imperative to introduce new technologies into road statistics, analysis, and application. Technology always comes from demand integration and planning. In the static and dynamic vehicle data of road traffic, after collecting the image data of original road traffic vehicles, license plate recognition [15] is carried out, which is written into the database, and then the statistics and analysis are consistently operated. Based on the direction of integration and database induction, it can be effectively analyzed and applied in issue of road traffic safety. Due to fast development of the smart city application in the recent years, smart technologies have motivated much attention to challenge the problems of transportation safety [16] addressed from both academicians and practitioners. This meaningful topic is thus emphasized on this study. This research takes the smart city infrastructure-traffic as the research main axis, through digital (data), system connection, and establishment of information technology (IT) infrastructure management, the sensing, analyzing, and integrating data are conducted in order to establish dynamics smart city. Figure 1 shows a schematic diagram for the smart city.

### 2.2. The IVS And CCD with Applications

Regarding IVS, it has taken as a basic tool for some security-related issues for controlling transportation surveillance system for vehicles in motivating the demands of smart cameras; therefore, its application of monitoring transport vehicles has become an interesting and promising lesson and challenge for contributing significant progress on road safety application. It not only storages large amounts of video data space into the cloud-based environment, but also has highly powerful computation ability. In particular, applications of the IVS and CCD of smart cameras irritate meaningful issues for different algorithms of surveillance systems with new functionalities [17] for the purpose of vehicle transportation safety. Given the above reasons, future directions and the lessons on such an issue is also highlighted on this study for searching a reliable means to study for it.

IVS and CCD are one of the key technologies of photography surveillance, which integrates the current problems faced by current video images with intelligent cloud traffic vehicle image and video tracking mechanism by using information technology [13]. From analog monitoring to digital monitoring to intelligent monitoring, IVS is different from the traditional image monitoring system that requires monitoring personnel to keep a close eye on the monitoring image, which has a new image analysis technology. Besides the core component CCD as the basic structure, IVS also has the basic ability to conduct cross-comparison analysis of “objects,” “events,” and “conditions” [2], to determine whether a specific object triggers an event, and then make an active response in the first time in accordance with the set conditions, which is equivalent to endowing the monitoring system with artificial intelligence and can identify the license plate and judge the behavior by itself, automatically detect abnormal situations, and immediately notify. Looking through the technical development of image surveillance, in addition to focusing on high-resolution and high-quality monitors, the future will attach importance on data identification processing and remote monitoring to meet the widespread and diverse needs of image surveillance. After the images are automatically detected and captured by real-time images, CCD uses the neural network and mathematical algorithm to capture the license plate on the image captured by the camera and to instantly recognize the letters and numbers on the license plate [18,19] for storage or other control purposes.

## 3. Research Methodology

This section introduces the methodology of designing a surveillance system of vehicles for completing a prototype of reliable smart city, including license plate recognition algorithm, framework of research, and the proposed ICTVSS model with experiencing a smart city application.

### 3.1. License Plate Recognition Algorithm

The key algorithms for dynamic license plate recognition in this study are divided into two categories: An improved combination of active contour algorithm and an improved differential algorithm. These two kinds of algorithms solve numerical and non-numerical calculus through numerical calculus program. The improved difference algorithm and improved combination of active contour algorithm are constructed on the difference among individuals in a group [20], and the differences between individuals are added to other individuals to observe whether the difference can bring positive results so as to obtain the evolutionary advantages. When the vehicle is running, the dynamic license plate recognition algorithm can start from an initial state and initial input, and it finally produces output and stop at a termination state after a series of limited and clearly defined states [21]. Image segmentation is a very important subject in the analysis and processing of digital image data for mobile object detection technology, especially for mobile object detection; it requires mature segmentation technology to clearly segment the foreground and background [22] and adds new cloud architecture idea with the hope to achieve better results. The two algorithms are described as follows.

#### 3.1.1. Improved Differential Algorithm

Regarding improved differential algorithm [22], it can be formatted as the following procedures and parameter setting step by step.

1. Initialization: Set parameter value.
2. Mutation: Randomly select three variable vectors (*Xr1*, *G*), (*Xr2*, *G*), and (*Xr3*, *G*), and obtain a donor vector (*V_i_*, *G+1*) through mutation weighting factor (*F*). The equation is defined as follows:
  *V_i_*, *G+1*= (*Xr1*, *G*) + *F* (*Xr2*, *G* - *Xr3*, *G*).
3. Crossover: Trial vectors are generated by crossover of synthetic vectors with target vectors *X_i_* and *G* selected from the group. In the *jth* iteration, the trail vector *U_i_*, the *i-th* component of *G* are composed of the target vectors (*X_i_*, *G*) or the donor vector (*V_i_*, *G*) and it can be formatted as follows:
  $$U_{j,i,G+1}=\{\begin{array}{cc} V_{j,i,G+1} & \mathrm{if}\;\mathrm{rand}\leq \mathrm{CR}\\ X_{j,i,G} & \mathrm{if}\;\mathrm{rand}>\mathrm{CR} \end{array}$$
4. Selection: Fitness function is used to select which of the trial vector and target vectors (*X_i_*, *G*) can enter the next iteration to become the target vector of the next iteration.
5. Activation: Operator activation strategy is introduced in order to give the algorithm a chance to mutate again. Activation promotes the algorithm to explore object space more widely and effectively. At the same time, activation strategy mechanism is used to avoid the problem of solution vectors falling into regional variability.
6. Iterative: An activity that repeats the feedback process, usually for the purpose of approaching the desired goal or result. Each repetition of the process is called an “iteration” and the result of each iteration obtained can be used as the initial value of the next iteration.
7. Mutation weighting factor: The mutation factor determines the amount of disturbance obtained in each iteration. The larger the mutation factor is, the amplitude of beating is larger in searching; at the same time, the group can obtain the optimal solution out of the region, but its convergence speed is relatively affected and slowed down. Conversely, if a smaller *F* value is set, the convergence speed increases, and the possibility of falling into the optimal solution in the region raises.

#### 3.1.2. Improved Combination of Active Contour Algorithm

For improved combination of active contour algorithm, the edge detection and dynamic contour model algorithm continuously tracks the change of object contour to be observed through the CCD camera, and the position information of object contour can be revised and updated at any time when the dynamic vehicle process changes. The improved combination of active contour algorithm consists of internal module formula and external module formula, and their format is specified and formed in the following steps.

1. Internal Module Formula

(1) The internal energy of contour curve in dynamic contour model algorithm is defined as the energy calculated by each single control point on the contour [19], representing the dependence of the distribution of control points on the contour curve.
(2) Expression: $E_{int}=\left(\alpha \left|V_{s}\left(S\right)\right|+\beta \left|V_{ss}\left(S\right)\right|\right)/2$.
(3) Where *α* and *β* are the parameters of the first and second terms, which mainly maintain the linearity and smoothness of the contour. The smaller the *α*, the lower the contour continuity; the smaller the *β* is, the smoother the contour becomes.
(4) In addition, $\left|V_{s}\left(S\right)\right|\;\mathrm{and}\;\beta \left|V_{ss}\left(S\right)\right|$ are the first-order differential (slope) and the second-order differential (slope change rate) of the control point *V_i_* of contour curve. Their equations are presented as follows.
  $\left|V_{s}\left(S\right)\right|=\left|\frac{dv_{i}}{ds}\right|=\left|V_{i}-V_{i-1}\right|$,
  $\left|V_{ss}\left(S\right)\right|=\left|\frac{d^{2}v_{i}}{ds^{2}}\right|=\left|V_{i}-2V_{i}+V_{i+1}\right|$.

2. External Module Formula

(1) The definition of external contour curve is calculated by image information, and the image gradient change at the position where the object contour appears is larger than that of its adjacent region.
(2) Expression: $E_{ext}=\gamma \left|G\left(x,y\right)\right|$.
(3) The above formula |G(x,y)| is the gradient value of the image, and *γ* is the controllable parameter. The main function of the external module is to cause the original contour to move and approach the image features.
(4) The gradient value obtained in this study is the Sobel operator, which uses the horizontal shielding *G_x_* and the vertical shielding *G_y_* to operate on the source image *I*(*x*, *y*). They are formatted as follows:
  *G_x_*(*x*,*y*) = $\left[\begin{array}{ccc} 1 & 2 & 1\\ 0 & 0 & 0\\ -1 & -2 & -1 \end{array}\right]$ *I(*x*,*y*),
  *G_y_*(*x*,*y*) = $\left[\begin{array}{ccc} 1 & 0 & -1\\ 2 & 0 & -2\\ 1 & 0 & -1 \end{array}\right]$ *I(*x*,*y*),
  *G*(*x,y*) = $\sqrt{G_{x}^{2}+G_{y}^{2}}$
(5) Automatic convergence determination: In the past, in the operation of dynamic contour model, it is mostly necessary to determine whether the contour is converged or not by manual inspection, and to adjust the supervisory formula of the number of convergence operations to determine. However, determination often needs to consume a great dealt of manpower and cannot meet the requirements of system automatic observation. In order to make the contour to have better convergence effect in the dynamic contour model algorithm, the algorithm is used to perform the change of control points on the contour to control the number of only slight change points, so as to achieve the purpose of automatic determination whether the contour has been converged or not automatic observation by calculating the object and contour area.

### 3.2. Research Framework

Due to a negative effort to deal with the poor quality of surveillance equipment and inefficient management, this paper intends to study the possibility of establishing an “mechanism and application of intelligent cloud-based transportation vehicle image tracking and evaluation” and the image tracking analysis based on the concept of intelligent monitoring that meets the practical demands of a modern city. The idea was centered on the performance of dynamic identification of license plate. The image of a license plate was digitally converted and transmitted to an integrated cloud-based database management system for classification, management, analysis, and application, as well as to improve the capability of digital monitoring for a metropolis. The existing license plate identification algorithm was studied and developed into an improved differential algorithm [23,24,25] and improved combination of active contour algorithm. Based on this combination, a smart monitoring and dynamic license identification algorithm was realized specifically for a smart city.

**Figure 2:**
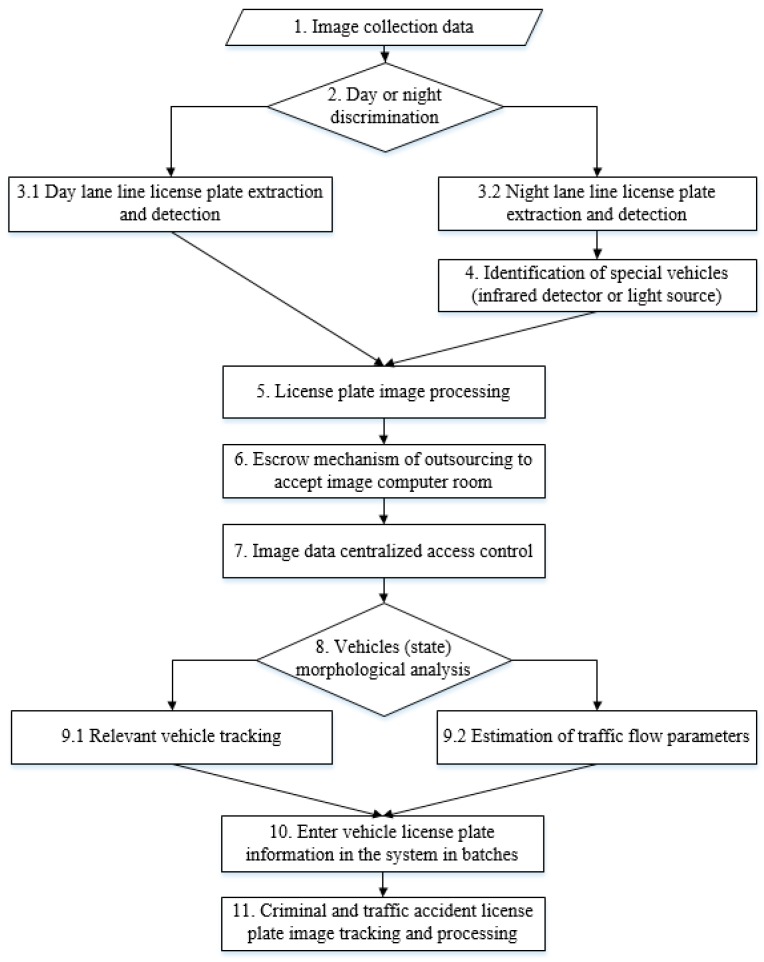
Flowchart of the proposed ICTVSS model in modularization steps.

In addition to using the important technology of IVS and CCD in expert interviews, this study focuses on how to improve the requirements of dynamic image capturing and fast recognition, and a set of smart city intelligent surveillance dynamic licence plate recognition algorithm based on a cloud-based platform is developed. According to expert interviews, the effectively control dynamic license plate recognition algorithm [21] should be based on the integration of the monitor equipment from forward to backward rather than importing it from backward to forward. In other words, the monitor image is clear, the relative recognition rate is high, and the availability of data analysis written into database is bound to increase. Thus, the proposed ICTVSS model is fully addressed. Figure 2 presents flowchart of the proposed ICTVSS model in 11 modularization steps for the prototype of smart city.

### 3.3. The Proposed ICTVSS Model for a Smart City Application

The paper implements the proposed ICTVSS model in determining the theoretical and technical steps of modularizations, as follows.

#### Step 1. Collect images data from camera: 

**Figure 3:**
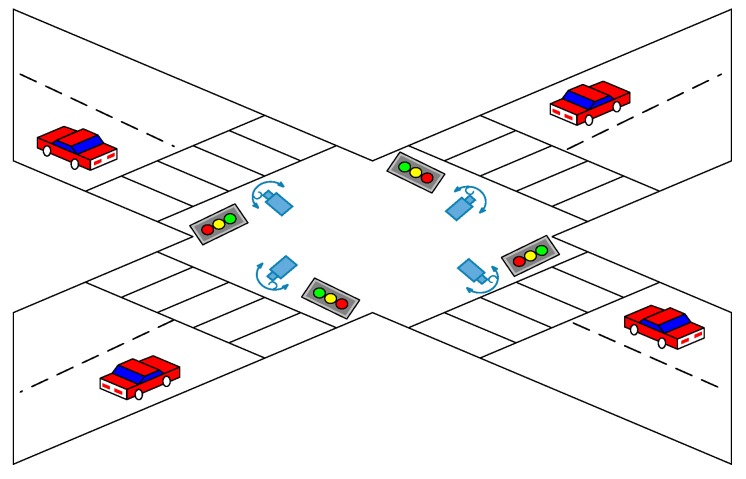
A schematic diagram for the camera erection.

Initially, this step will use the example data that can be collected from the cameras that are set up on the main road in New Taipei City of Taiwan to determine the practical background for CCD issues. The main road can be selected from the potentials of the first 10 accident-prone road segments and intersections for improvement purpose. Figure 3 shows a schematic diagram for the camera erection in the 10 key roads. The collected data will be recorded and analyzed in the future to study the real-life application case used in the structure of developing smart city.

#### Step 2. Judge day/night environment:

Accordingly, it is needed to first judge day or nighttime automatically according to the real environment in order to adjust adequately the optimal parameter to optimize the video effect. Importantly, the daytime should be in using inconstant-light sources for information retrieval from context features; conversely, the constant-light sources are used in the nighttime with the same purpose for the next step for respective characteristics of image processing.

#### Step 3. License plate localization and detection:

This step is divided into two sub-steps for accurately executing license plate localization and detection in order to identify efficiently the data of day or night of environment, respectively. The two sub-steps are introduced as follows.

#### Step 3.1. Identification of daytime:

**Figure 4:**
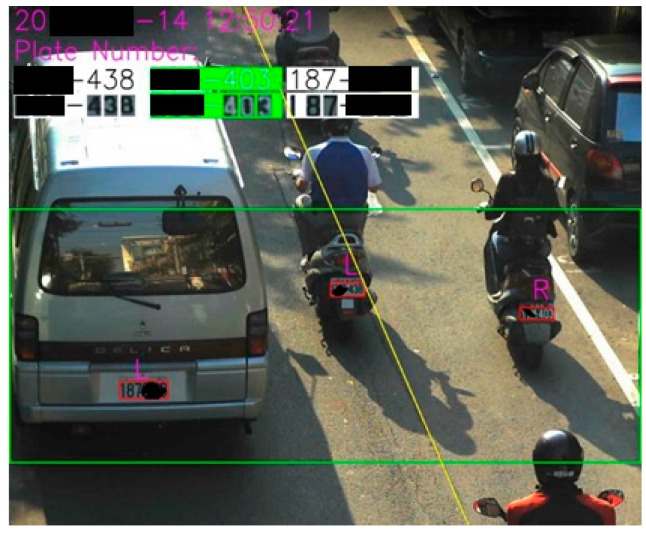
A schematic diagram for detecting the identification of daytime objects.

Based on the theoretical concepts of the study of El-Said [26], to use the identification of license plate localization and detection for daytime objects is executed for different and respective traffic lanes for extracting accurately features of digital video image from transportation tools. Figure 4 shows an example of schematic diagram in the identification of daytime objects for a real environment in Taiwan.

#### Step 3.2. Identification of nighttime:

**Figure 5:**
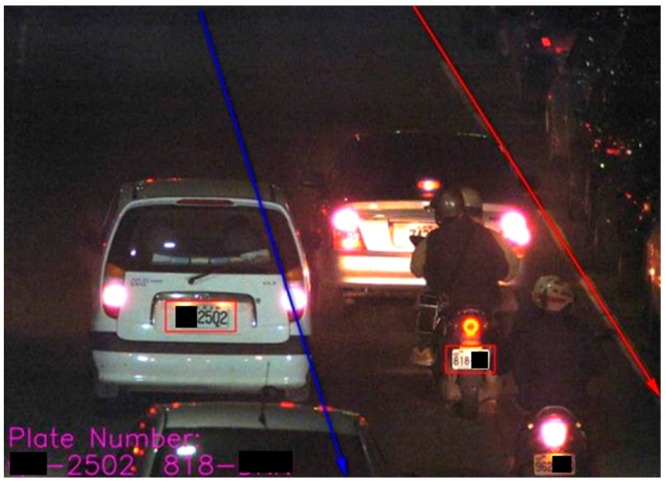
A schematic diagram for detecting the identification of nighttime objects.

Correspondingly, it has some difficult for extracting adequately the identification of nighttime image from moving objects due to its insufficient light sources when comparing to daytime condition; thus, it needs more enough technique processing to address this problem, such as the study of Kannan [11,26,27]. Figure 5 shows a case of schematic diagram in identifying accurately the nighttime objects.

#### Step 4. Identification for special case of vehicle:

**Figure 6:**
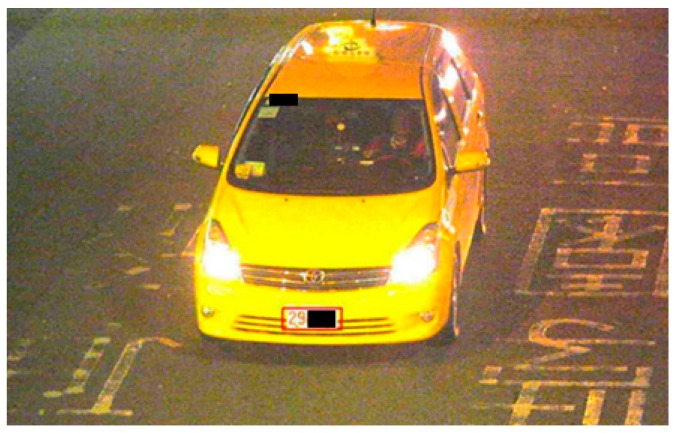
A schematic diagram for detecting the identification of special case of vehicle.

From literature review of the study in Zheng et al. [27], an effective method of using infrared detector and light source to detect moving points on objects is employed with both purposes of the verification and examination from special pattern images for extracting the characteristics on the special case of vehicle, such as a business taxi. Figure 6 lists a schematic diagram for identifying the special case of vehicle by a technique of infrared detector [26].

#### Step 5. Image processing of license plate:

There are five procedures for the image processing of license plate, including orderly image processing, license plate localization, license plate processing, character segmentation, and identifying recognition. In detail, the first sub-step is to process image extraction [20] from features targeted and accordingly locate and circle the position of license plate [28]. Next, this sub-step uses the processing algorithm in the study of Weijian and Zhou [29] for the following purposes of character segmentation application and license plate recognition [30,31].

#### Step 6. Construction of co-location for collecting images database:

This step mainly constructs an images database for images data collection by a co-location mechanism rule. Building an information system room often encounters many problems, such as security of the building where the computer room is located, space expansion, power shortage, maintenance manpower, bandwidth limitation, etc. Professional division of labor has become the operational model of modern public institutions. The escrow agency only needs to place the services or archives to be shared in the Internet data center (IDC) [32,33,34]. Without the limitations of bandwidth due to passing through the switch, it can directly transmit various services to the demand agencies so that the service quality can be further improved. IDC room provides constant temperature and humidity air conditioning environment, safe firefighting equipment, 24-hour access control, and duty personnel, as well as professional chassis and stable power monitoring.

#### Step 7. Centralized control for using the digitalized images:

This system uses a cloud-based structure to centralized control for the data of digital images extracted from IDC by a regulator based on various types of permission (or authority) to access to relevant data from surveillance images. In the case example, the user can extract specific vehicle image based on different requirements of the road section, expected date, and time interval whenever necessary.

#### Step 8. Analysis result of vehicle status:

From the collected images, it is clear that the vehicle status for the analysis result of moving vehicle can be immediately and accordingly recorded, recognized, and monitored from the consequences of above steps. 

#### Step 9. Special process for the specific vehicle status: 

This step is also categorized into two sub-steps for the following main activities of examination and verification for addressing the specific vehicle status, respectively.

#### Step 9.1. Track of target vehicle:

**Figure 7:**
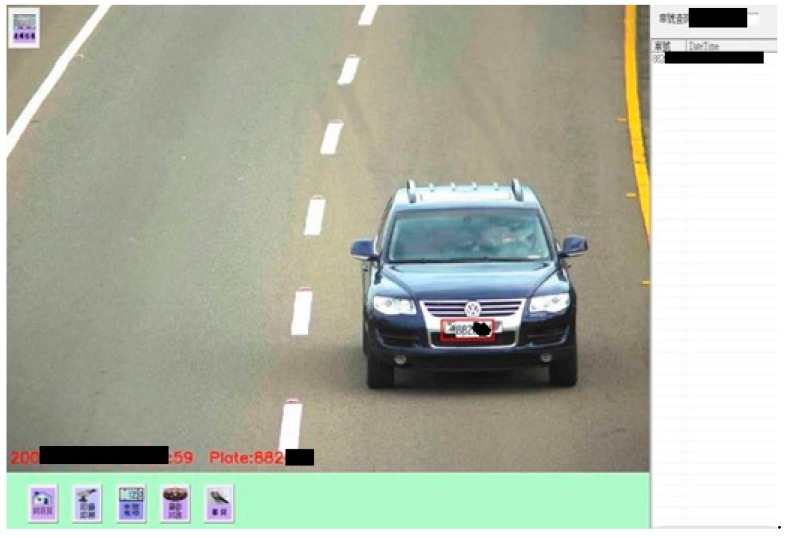
A schematic diagram for detecting the track of target vehicle.

The system can first track and examine the target vehicle by keying a license plate number for knowing adequately its localization and running paths from IDC database. Figure 7 presents a real case for the right result of tracking a specific vehicle. (Note: it is clear that the license plate for the specific vehicle can be correctly identified by the proposed ICTVSS model from Figure 7.)

#### Step 9.2. Identification of traffic flow: 

**Figure 8:**
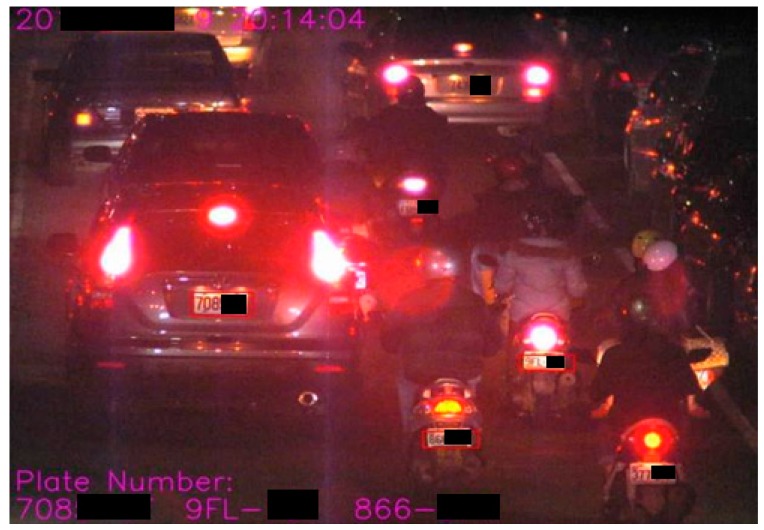
A schematic diagram for detecting the obstruction case.

This step can accordingly identify and verify the feature density for detecting the obstruction or smooth by tracking a slow or fast-moving flow of traffic image for license plate recognition. Figure 8 presents an obstruction case from the collected images focused on recognizing license plate.

#### Step 10. Batch import for the identified license plate:

Based on the requests of a specific regulator, the data for the identified license plate can be imported into the motor vehicle supervision system or the police supervision system by batch job for the purposes of examination and monitoring; and then the related data for the name of driver or vehicle owner, vehicle information, type, and manufactured year can be easily acquired for the purpose of screening geopolitical relationship to achieve chasing people by cars or chasing cars by people.

#### Step 11. Follow up the traffic accident vehicles:

Lastly, the vehicle having traffic accident or a case of criminal responsibility will be followed up connectively from the collected images processing for tracking and learning associativity of a specific vehicle to get complete dynamic vehicle trajectory.

## 4. Empirical results and analysis

**Figure 9:**
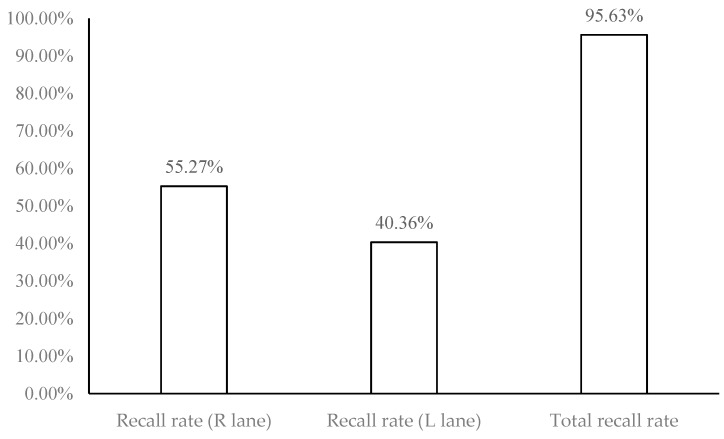
The recall rate and total recall rate for vehicle license plate.

**Figure 10:**
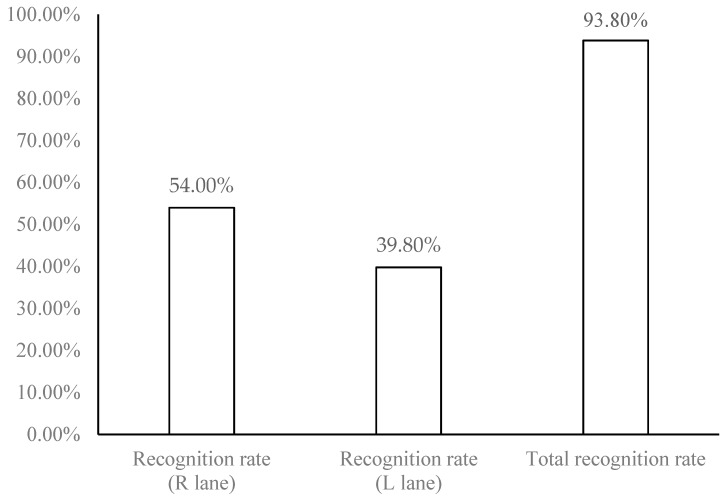
The recognition rate and total recognition rate for vehicle license plate.

The relevant literatures on license plate recognition all focus on static or semi-static license plate recognition. In real experiments of this study, two fixed 3 megapixel high-definition cameras were set up at the junction of Zhongzheng Rd., Zhonghe Dist., New Taipei City, Taiwan to connect Jingping Rd. They were used to take dynamic road monitoring images, including two kinds of traffic videos during the day and at night. Analyze and judge that they are daytime or night images, and find out the moving vehicles. After obtaining the vehicle license plate videos, immediately record and identify them, through dynamic continuous photography, 25–30 images per minute are identified, and the used license plate location information is not taken out manually. Accordingly, the license plate recall rate in this study refers to through license plate recognition algorithm, the dynamic vehicle obtained by the intersection photography, and it can be used to identify the image files, known as the recall rate of license plate. The recognition rate refers to the image files obtained by the intersection photography after the vehicle license plate is recalled, and the recognized image files are converted into data through morphological operation and write into the database, known as the recognition rate of accuracy. The empirical results of this study take the 20 minutes video recording of the road section mentioned-above as an example. 711 dynamic vehicles were collected, the numbers of license plate recall reached 680 vehicles, and the numbers of correct recognition was 667 vehicles. The analysis results experienced are shown in Table 2: Vehicle identification results for recall and recognition of license plates. The details for the results are descripted as follows:

1. The 680 vehicles of license plate had recalled.

(1) Inside (R) lane: 393 vehicles recalled / 711 dynamic vehicles collected = 55.27% (recall rate of license plate).
(2) Outside (L) lane: 287 vehicles recalled / 711 dynamic vehicles collected = 40.36% (recall rate of license plate).
(3) The total recall rate of vehicle license plate is 95.63% (55.27% + 40.36%), as shown in Figure 9: Recall rate of license plate and total recall rate of license plate.

2. The 667 vehicles were correctly recognized.

(1) Inside (R) lane: 384 vehicles recognized / 711 dynamic vehicles collected = 54.00% (recognition rate).
(2) Outside (L) lane: 283 vehicles recognized / 711 dynamic vehicles collected = 39.80% (recognition rate).
(3) The total recognition rate is 93.80% (54.00% + 39.80%), as shown in Figure 10: Recognition rate and total recognition rate.

**Figure 11:**
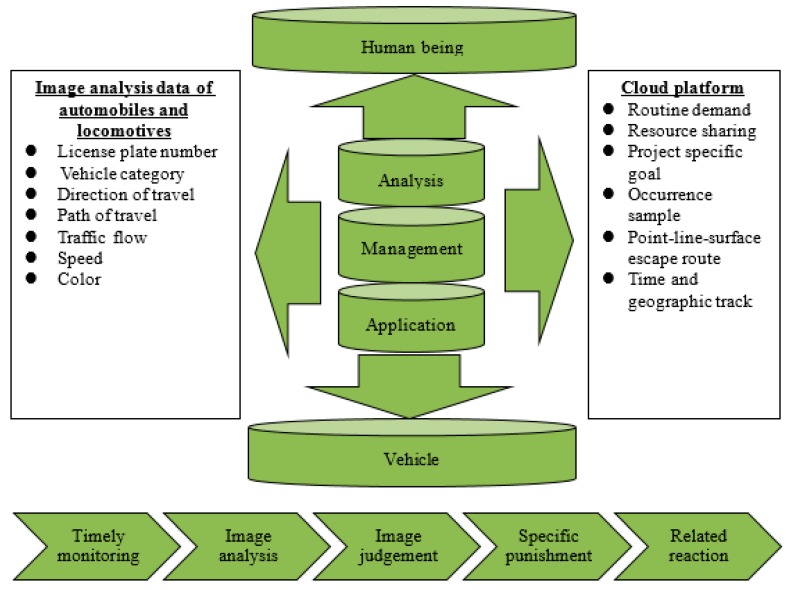
Expected benefits diagram.

After the digitalization of license plate images, the derivative benefits can be expected, as shown in Figure 11: Showing expected benefits diagram. Using high-resolution digital cameras to improve the picture quality and expand the scope, effectively monitoring and regularized database in the background can greatly reduce personnel re-access dispatch, data search, and other work or resource waste, so as to make more effective allocation and application of manpower. In addition, all parameters can be integrated, such as license plate, vehicle type, and automatic analysis. After parameter interpretation, they can share and alert mutually to prevent people from being hurt by personal and property.

## 5. Academic Implications and Managerial Implications

The difference between this study and related literature is that full dynamic license plate recognition, and has been verified. The initial results can be used as reference for academic and practical contributions, hoping that this new technology can be the beginning of a whole new technology. The academic and practical contributions from the empirical results are described as follows.

1. Academic contributions:

(1) In the non-constant light source and multi-dynamic complex scene environment at the intersection, through the high-resolution camera monitor, the improved differential algorithm and the improved combination of active contour algorithm developed for dynamic license plate recognition were used to overcome the complex environment of the intersection, so as to achieve multi-objective to accurate license plate positioning, segmentation technology, anti-noise, and light source processing for license plate images, and carry out effective algorithmic image convergence and multi-objective locking for license plate recognition accuracy.
(2) After collecting the identifying license plate, it can analyze the ratio of traffic flow to road capacity through big data according to the vehicle traffic and vehicle attributes, and provide statistical analysis and application of data for the traffic authorities, relevant units, and academic institutions as an important reference for traffic safety, engineering improvement, and advocacy as well as the basis for future big data analysis.
(3) Although there are some studies that focus on dynamic license plate recognition, such as the literature of Ho et al. [35], there is less literature data on this issue when compared to general license plate recognition. Most papers and literature discussed the mathematical problems of how to deal with static or semi-static license plate recognition through limited steps. The difference of this paper is that this research developed advanced dynamic license plate recognition algorithms, and testifies them one by one. Although the identification data is not so much, it can provide future direction for development and research.

2. Practical contributions:

(1) At present, most of the road and intersection photographic surveillance systems are too low in image quality, blurred in image, time-consuming for manpower access, and difficult to prove, so the obtained images cannot be fully utilized. Based on the test results and the integration of the equipment from the front to the back, this study started from the key technologies of camera surveillance image—IVS and CCD [36]. The empirical results show that the image pixel and feature quality are clear and can be used to monitor the state of traffic events clearly, and be transplanted to practice to further understand and analyze, in response to the popularity and diverse needs of image monitoring. Clear source can improve the recognition rate, and the availability of data analysis written in database must be accurate.
(2) Using the dynamic license plate recognition algorithm, the empirical results show that the total recall rate and the total recognition rate of license plates are up to 95.63% and 93.80%, respectively, and the related images can be queried in real time according to the set conditions. The cloud planning management mechanism enables the digital file management, not only the video image access (check). High-resolution image can clearly restore and detect the dynamic trajectory of automobile, locomotive vehicles, and the images of traffic accident causes, to provide an important basis for the follow-up litigation, claims, and liability prosecution after providing relevant experts.
(3) Smart city is an ecological circle connected by planning and management, infrastructure construction, and citizen satisfaction, which can promote sustainable economic growth and become a smarter city in the future. Observing the different patterns and trends of the development of “smart cities” at home and abroad, in fact, cloud management, massive database integration, and computer room co-management have become the essential foundation. At the same time, the current situation of telecom operation to implement wireless Internet access and the popularity of smart phone with the public is rapidly changing the appearance of urban public services. The development of smart cities can inevitably be integrated into the application of various technologies, including cloud application services, government open data model, etc., which all can improve the efficiency of urban management.

## 6. Conclusions

In terms of algorithm, the existing license plate recognition algorithms are modified and combined with the improved differential algorithm and the improved combination of active contour algorithm. A set of smart city for intelligent monitoring dynamic license plate recognition algorithms is developed. In order to realize the ideal of smart city, this study has proposed an advanced combined algorithm for vehicle license plate recognition to improve traffic safety, which is named as ICTVSS model. For the research and verification in this study, the Zhongzheng Road in Zhonghe District in New Taipei City, Taiwan is a frequent traffic accident, and that is as the proving ground. It is also ensured that effective identification of license plates with the help of high-definition cameras is experienced. Through the shooting lens of 20 minutes by the ICTVSS model, that is total of 711 vehicles license plate photos and carried out research and analysis.

The empirical analysis from the experiment results achieves accurate multi-objective license plate positioning, segmentation technology and license plate image anti-noise, unsteady light source, and constant light source processing through the integration of high-resolution camera monitor in complicated scene environment, and with cloud planning management mechanism. Productively, the proposed ICTVSS model is performed well and satisfied with the empirical results. More importantly, it is indicated that the integration of cloud-based panning and management mechanism can contribute to the achievement of tracking vehicles, which escapes civil and criminal cases, clarification of responsibility in traffic accidents, identification of causes, and traffic flow statistics. Moreover, digital file management was enabled as the basis of future big data analysis, not only to provide the video image access (check), but also to jointly build a modern city of traffic safety and quality of life.

Lastly, although the proposed ICTVSS model of this study has merits and contributions with good performance on application of the advanced license plate recognition for the purpose of developing a smart city for citizens, it is necessary to provide a room to explore the benefits of joint and use about more references, such as a well-known technique (i.e., Monte Carlo simulation) about different Kalman filters, particle filtering methods/techniques, for filtering features, localization, and tracking with application for relevant urban mobility and urban management in the subsequent research. Future research can be focused on the following five directions. (1) Three studies can be addressed for understanding a variety of Monte Carlo simulation. First, for achieving an effective urban management, Watanatada and Ben-Akiva [37] had developed a comprehensive urban travel demand prediction model of urban passenger for policy-sensitive sketch planning by Monte Carlo simulation. Next, Martino et al. [38] designed a sequential Monte Carlo scheme and integrated interacting parallel particle filters for a global estimator of the variable of interest and an approximation of the posterior density for the application context of urban mobility. Third, Mitropoulos et al. [39] combined fuzzy logic and Monte Carlo simulation for the sustainability assessment of urban transportation vehicles to measure the applicability of the method to selected indicators in ranking the sustainability performance of vehicles. Furthermore, (2) alternative for a more real case of image processing evaluation on other road section for the dynamic license plate recognition is needed. (3) Add more testing data on the same section for further verifying the study performance. (4) Add more analysis criteria for further measuring the proposed ICTVSS model. (5) Other models can be joined into a hybrid model to further examine and justify the proposed model. 

## Figures and Tables

**Table 1:** Information of causes and casualties of road traffic accidents in 2003–2018.

Year	Causes in Cases						Casualties in Persons		
	Grand Total	Automobile Driver Faults	Mechanic Malfunctions	Pedestrians Faults	Traffic Control Defects	Others	Grand Total	Deaths	Injuries
2003	120,223	117,906	412	1678	123	104	159,021	2718	156,303
2004	137,221	134,383	446	2036	140	216	181,742	2634	179,108
2005	155,814	152,408	443	2521	198	244	205,981	2894	203,087
2006	160,897	157,428	490	2571	182	226	214,316	3140	211,176
2007	163,971	160,646	474	2374	228	249	219,500	2573	216,927
2008	170,127	166,728	561	2380	186	272	229,647	2224	227,423
2009	184,749	180,806	709	2676	230	328	249,086	2092	246,994
2010	219,651	215,153	749	2957	286	506	295,811	2047	293,764
2011	235,776	230,892	811	3137	307	629	317,318	2117	315,201
2012	249,465	244,306	856	3119	350	834	336,122	2040	334,082
2013	278,388	272,541	930	3635	348	934	375,496	1928	373,568
2014	307,842	301,685	985	3843	309	1020	415,048	1819	413,229
2015	305,413	299,003	946	3895	305	1264	411,769	1696	410,073
2016	305,556	299,357	936	3572	350	1341	405,510	1604	403,906
2017	296,826	291,073	797	3401	357	1198	395,715	1517	394,198
2018	320,315	313,974	922	3625	376	1418	429,542	1493	428,049

**Table 2:** Vehicle recall and recognition results of license plates.

Video	Traffic Condition	License Plates Recall Rate		Recognition Rate		Total Recall Rate of Vehicle License Plates	Total Recognition Rate
1	Smooth	R lane	L lane	R lane	L lane		
		55.27%	40.36%	54.00%	39.80%	95.63%	93.80%
		680 vehicles recalled		667 vehicles recognized			
